# Inhibition of androgen receptor by decoy molecules delays progression to castration-recurrent prostate cancer

**DOI:** 10.1371/journal.pone.0174134

**Published:** 2017-03-17

**Authors:** Jae-Kyung Myung, Gang Wang, Helen H. L. Chiu, Jun Wang, Nasrin R. Mawji, Marianne D. Sadar

**Affiliations:** Genome Sciences Centre, British Columbia Cancer Agency, Vancouver, British Columbia, Canada; Florida International University, UNITED STATES

## Abstract

Androgen receptor (AR) is a member of the steroid receptor family and a therapeutic target for all stages of prostate cancer. AR is activated by ligand binding within its C-terminus ligand-binding domain (LBD). Here we show that overexpression of the AR NTD to generate decoy molecules inhibited both the growth and progression of prostate cancer in castrated hosts. Specifically, it was shown that lentivirus delivery of decoys delayed hormonal progression in castrated hosts as indicated by increased doubling time of tumor volume, prolonged time to achieve pre-castrate levels of serum prostate-specific antigen (PSA) and PSA nadir. These clinical parameters are indicative of delayed hormonal progression and improved therapeutic response and prognosis. Decoys reduced the expression of androgen-regulated genes that correlated with reduced *in situ* interaction of the AR with androgen response elements. Decoys did not reduce levels of AR protein or prevent nuclear localization of the AR. Nor did decoys interact directly with the AR. Thus decoys did not inhibit AR transactivation by a dominant negative mechanism. This work provides evidence that the AR NTD plays an important role in the hormonal progression of prostate cancer and supports the development of AR antagonists that target the AR NTD.

## Introduction

The prostate is an androgen-dependent tissue that requires androgen for the growth and survival of epithelial cells. Androgen receptor (AR) is a transcription factor that mediates the effects of androgen. It is composed of functional domains that include a C-terminal ligand-binding domain (LBD) that contains transactivation function-2 (AF-2), a DNA-binding domain (DBD), and an N-terminal domain (NTD) that harbors AF-1 with two transcriptional activation regions [1]. All current approved hormonal therapies for prostate cancer aim at preventing activation of AR through chemical or surgical castration and intervention with antiandrogens that competitively bind to the LBD of the receptor. These hormonal therapies include LHRH analogues, enzalutamide and other antiandrogens, and abiraterone. Initially, prostate cancer responds to these therapies. However, inevitably the disease will become lethal castration-recurrent disease. AR is suspected to continue to drive castration recurrent disease. The transcriptional activity of AR is dependent upon functional AF-1 [1] thereby providing rationale to develop approaches that inhibit AR by targeting its NTD.

In 2007, the first *in vivo* proof-of-concept for AR NTD as a novel therapeutic target was provided using copies (decoys) of the AR NTD residues 1–558 (AR_1-558_) [2]. In non-castrated hosts, these decoys reduced tumor incidence, decreased tumor growth and serum PSA levels [2]. Here we provide evidence that lentiviral delivery of decoys to mice bearing established prostate cancer xenografts inhibited hormonal progression to castration-recurrence as well as investigated possible mechanisms through which decoys exert their activity.

## Materials and methods

### Animals and cell culture

Male NOD-SCID mice were obtained from the Animal Research Center of the British Columbia Cancer Agency. All animal studies conformed to the relevant regulatory and ethical standards. Analgesic (Metacam) and anaesthesia (isoflurane) were used and all efforts were made to minimize suffering. The University of British Columbia Animal Care Committee approved all experiments involving animals (Permit Number A03-0260). LNCaP cells (from Dr. Leland Chung, Cedars-Sinai Medical Center, Los Angeles, CA) were routinely maintained in RPMI 1640 supplemented with 5% (v/v) FBS (HyClone, Logan, UT). LNCaP cells that stably express decoy AR_1–558_ have been described [2]. The synthetic androgen (R1881) was purchased from Perkin–Elmer (Wellesley, MA) and forskolin was purchased from Calbiochem (La Jolla, California, USA).

### Plasmids

His-tag expression plasmids for AR_1-558_, AR_1-233_, and AR_392-558_ plasmids were made by polymerase chain reaction (PCR) amplification of the nucleotides of the cDNA corresponding to the amino acids 1–558, 1–233, and 392–558 of the human AR and cloning the products into the BamHI site of pcDNA3.1/His^©^A plasmid (Invitrogen, Carlsbad, CA). The human AR_1-558_ decoys and lentivirus plasmids have been described [2]. The PSA (-630/+12)-luciferase reporter contains the promoter region with two well-characterized AREs [3,4].

### Lentivirus delivery and castration in mice

LNCaP xenografts were established subcutaneously (s.c.) in the flanks of 6-week-old male NOD-SCID mice [2]. The lentivirus particles were prepared by using the ViraPower expression system (Invitrogen) as previously described [2]. When tumors averaged approximately 50–100 mm^3^ in size, the animals were randomly divided into four groups (Mock media, GFP, GFP-AR_1–558_, and AR_1–558_). Treatment consisted of injections every 5 days with 1–2 x 10^7^ particles for GFP-AR_1–558_ and AR_1–558_ and 1x10^8^ particles for GFP for the duration of the experiment. Tumors were measured weekly. Castration was performed under anesthesia by making a small incision in the scrotum to remove each testicle after ligation of the cord. At 5 days after the last inoculation, mice were sacrificed, and the tumors and major organs were excised and prepared for immunohistochemistry and Western blot analyses.

### Serum PSA levels

Blood samples were obtained from mice weekly before and after castration. Serum PSA levels were determined by an enzymatic immunoassay kit with a lower limit of sensitivity of 0.2 μg/liter (Abbott IMX, Montreal, QC, Canada).

### Immunohistochemistry

Tissue sections (5 μm) were blocked in immunohistochemistry solution (Immunovision Technologies, Brisbane, CA) and immunostained with anti-AR LBD (C-19, Santa Cruz Biotechnology, Santa Cruz, CA), or anti-AR NTD (441, Santa Cruz). The Vectastain^®^ ABC Kit (Vector Laboratories, Burlingame, CA) was used for detection. Peroxidase activity was localized with 3,3-diaminobenzidine, and the sections were counterstained with hematoxyline before dehydration and mounting. The slides were visualized using a Zeiss Axioplan 2 Microscope (Carl Zeiss, Toronto, ON, Canada).

### Western blot analysis

Whole cell protein lysates were obtained from frozen xenografts that were ground up in N_2_ (liquid) then homogenized using a polytron homogenizer in ice-cold RIPA buffer (40mM Tris-HCl, pH7.0/ 1mM EDTA/4%glycerol/10mM DTT/0.2%SDS/20mM Na Molybdate/50mM NaF/Complete™ protease inhibitors (Roche, Mississauga, ON, Canada). The protein lysates were treated with Albumin Depletion kit (Millipore). Protein concentrations were determined by RC DC assay (BioRad). Protein samples (40 ug) were loaded on a 10% polyacrylamide gel, transferred on Immobilon™ PVDF membranes (Millipore, Billerica, MA), and probed with anti-GFP (Santa Cruz), or anti-AR (PG21, Upstate) antibodies. Whole cell lystes from LNCaP cells transfected with the His-Tag plasmids were loaded on a 12.5% SDS PAGE and analyzed by Western blot analysis using anti-His-probe (H-15; Santa Cruz Biotechnology).

### GFP-AR transfection and microscopy

LNCaP cells (2.5 x 10^4^) that stably express vector or AR_1-558_ [2] were plated in 4 well chamber slides and transfected with 0.25ug GFP-AR plasmid DNA per well using lipofectin and treated with R1881 (10 nM) for 30 minutes. The cells were fixed in 4% paraformaldehyde (EM Sciences) for 30 minutes at room temperature and then washed several times with PBS. The slides were mounted with Vectasheild mounting medium (vector labs) and examined using a Zeiss Axioplan-2 Fluorescent microscope (Zeiss).

### Quantitative real-time PCR (qPCR)

Levels of expression of androgen-regulated genes were analyzed in LNCaP cells that stably express vector or decoy as well as in xenografts derived from mice. LNCaP cells were treated with R1881 (10 nM) for 24 hours and total RNA isolated in Trizol® Reagent (Life Technologies). For androgen-repressed genes, cDNA was generated using the high Capacity RNA-cDNA kit (Applied Biosystems). The appropriate cDNA dilution was mixed with gene-specific primers and Platinum SYBR Green qPCR Supermix–UDG with ROX. Transcript levels were measured using ABI 7900HT qPCR machine. Xenografts from mice with injected empty lentivirus vector, lentivirus vectors for AR_1–558_, GFP, and GFP-AR_1–558_ were flash frozen with liquid nitrogen prior to isolation of total RNA also with Trizol®. Oligo-d(T)-primed total RNAs (5 μg per sample) were reverse-transcribed with SuperScript III (Invitrogen). An appropriate dilution of cDNA and gene-specific primers were combined with iQ SYBR Green Supermix (Bio-Rad) and amplified in an iCycler iQ real-time PCR machine (Bio-Rad, Hercules, CA). All qPCR reactions were performed in triplicate. Ct (threshold cycle number) and expression values with standard deviations were calculated using the Gene Expression Macro for Excel (Bio-Rad). Real-time amplification was performed with initial denaturation at 95°C for 2 min, followed by 40 cycles of two-step amplification (95°C for 15 sec, 55°C for 30 sec). The results were normalized for GAPDH as housekeeping gene. Primer sequences for real-time PCRs were as listed in Table 1.

**Table 1:** Primer sequences for gene expression analysis using real-time PCR.

Gene	Forward (from 5' to 3')	Reverse (from 5' to 3')
CAM2KN1	ATTCTGTATGTTGCACCTTG	TTGAGACACAGGAACAATTC
GAPDH	CTGACTTCAACAGCGACACC	TGCTGTAGCCAAATTCGTTG
HMGCR	TCCCTGGGAAGTCATAGTGG	AGGATGGCTATGCATCGTGT
KLK2	TGTGTGCTAGAGCTTACTCTGA	CCACTTCCGGTAATGCACCA
KLK3	CCAAGTTCATGCTGTGTGCT	CCCATGACGTGATACCTTGA
MAF	CCGTCCTCTCCCGAGTTTTTC	ACACTGGTAAGTACACGATGCT
MMP16	ACCCTCATGACTTGATAAC	TCTGTCTCCCTTGAAGAAATAG
RHOU	CCCGTGAGACTCCAACTCTG	TGAAGCAGAGCAGGAAGATG
SAT1	CACTGGACTCCGGAAGGTAA	TCATTGCAACCTGGCTTAGA
SESN2	CTGACTACTTTACCAGCTTC	TACCAGGTAAGAACACTGATG
SLC44A1	TCAGTAAATCGCCTTATTCG	TTTTCCTTTCCTTTGAGCTG
ST7	CTGCTTATATTCTCTTGGCTG	GTCTATGTTGGGCTTCATAC
TMEM144	TTTCCAATAATCACTGCTGG	ATAAGGCTCCAGTCAAGATG
UGTB15	GCAAATCTCTACTTGACACATGG	CTTCTTGGTCATCCCAAAAC
UGTB17	ATTCTGCTCAAAATGAAGCC	CTGAGCTTCCTTATGTTTCAC

### Chromatin Immunoprecipitation Assay (ChIP)

LNCaP (1x10^6^ cells) stably transfected with vector or AR_1-558_ were plated in 10cm plates (Nunc, Rochester, NY). The next day the media was replaced with 9 ml of serum-free RPMI. The cells were treated for 30 mins with R1881 (10nM), cross-linked with 1% formaldehyde and harvested. The cells were lysed in SDS lysis buffer (1%SDS, 50nM Tris (pH 8.0), 10mM EDTA, Complete™ protease inhibitors), and sonicated. The extracts were used for immunoprecipitation with anti-AR antibody AR (C-19; Santa Cruz Biotechnology). PSA primers used for real time PCR with Sybr green qPCR kit from (Invitrogen) were: 5’-GCCTGGATCTGAGAGATATCATC-3’ (forward) and 5’-ACACCTTTTTTTTCTGGA TTGTTG-3’ (reverse) [5].

### Coimmunoprecipitation

LNCaP cells that stably express vector or AR_1-558_ [2] were cultured in RPMI 1640 culture medium supplemented with 5% FBS (v/v), 100 units/mL penicillin, and 100ug/uL streptomycin. For treatment with androgen, approximately 3x10^6^ cells were plated individually into 15cm tissue culture dishes. After 24 hours, LNCaP cells were incubated in serum-free medium for 48 hours, treated with 1nM R1881 for 3 hours and harvested. Proteins were extracted from harvested LNCaP cells and lysed in buffer containing 10mM Tris-HCl (pH 7.4), 1% NonidetP-40, 150 mM NaCl, 0.5% sodium deoxycholate, 1 mM EDTA, 10ug/ml leupeptin, 10ug/ml aprotinin, 1mM AEBSF, 2mM Na_3_VO_4_, 10mM beta-glycerophosphate, and Complete™ protease inhibitor cocktail (Roche). Whole cell lysates were centrifuged at 14,000rpm for 20min at 4°C and supernatant was used for immunoprecipitation. Pre-clearance was done by incubation with rabbit IgG (Santa Cruz Biotechnology) and Protein A/G-agarose beads (Santa Cruz Biotechnology, sc-2003) for 1hour at 4°C with rotation. Protein complexes were incubated with AR antibody(C-19) (Santa Cruz Biotechnology, sc-815) for 90 minutes at 4°C and pulled down with Protein A/G agarose beads. After overnight incubation, protein complexes were washed with lysis buffer three times, eluted with SDS-PAGE sample loading buffer and separated by SDS-PAGE. Western blotting was performed using anti-AR(441) (Santa Cruz Biotechnology, sc-7305) and the protein bands were visualized by chemiluminescence (Amersham Biosciences).

### Luciferase assay

LNCaP cells (3x10^5^ cells/well) were plated on 6-well plates (Falcon) containing RPMI with 5% FBS. After 24 hours, the medium was removed and transfection was performed by using Lipofectin^®^ Reagent (Invitrogen). The total amount of plasmid DNA was normalized to 3 μg/ well by the addition of pRc/CMV as the control plasmid with no promoter insert. The cells were cotransfected with PSA promoter-luciferase reporter gene and various portions of AR NTD or His-tag. Cells were treated with or without forskolin (50 μM) for 48 h. Cells were harvested in 1×Passive Lysis Buffer (Promega, Madison, WI, USA) and luciferase activity in the cell lysates was measured using the Luciferase Assay Reagent (Promega). The luciferase activity was normalized to the protein concentration by Bradford assay. The inhibitory effect of each ARN decoy molecule was calculated from the normalized luciferase expression to that of the His-tag control.

## Results

### Lentivirus delivery of decoy AR_1-558_ inhibits hormonal progression of established LNCaP tumors in castrated mice

**Fig 1:**
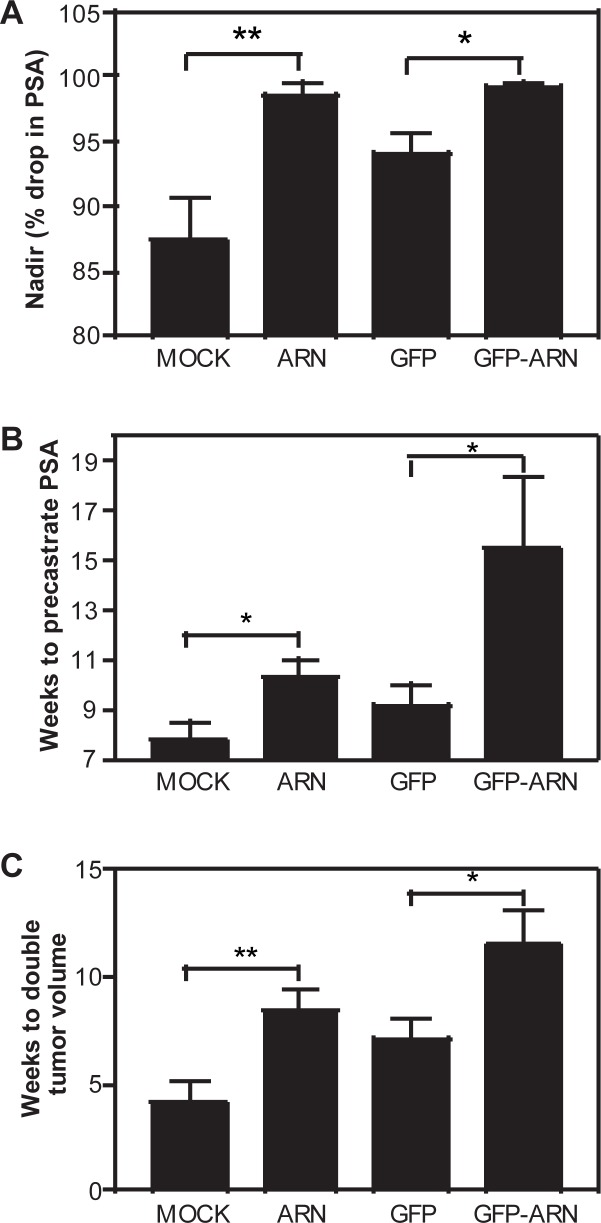
Lentivirus delivery of decoy AR_1-558_ delays the time to castration-recurrence. A, PSA nadir or percent drop in serum PSA levels in response to castration of mice bearing LNCaP xenografts and treated with lentivirus for mock (vehicle control), decoy AR_1-558_ (ARN), GFP, or GFP-AR_1-558_ (GFP-ARN). B, The time to reach pre-castration levels of serum PSA was doubled in animals injected with decoys. C, The time for the tumor volume to double was increased by decoys. Tumors were inoculated 5 days before castration and subsequently injected every 5 days until the duration of the experiment. Student t-test: *, *p*<0.05; **, *p*<0.01.

To determine the effects of decoy on the time to castration-recurrence, we delivered AR_1-558_ decoys by lentivirus to established LNCaP xenografts in combination with androgen ablation therapy (castration). Once the tumors were 50–100 mm^3^ in volume, the mice were randomly assigned into 4 groups that were: mock injection (vehicle only); decoy AR_1-558_; GFP; and GFP-AR_1-558_. Animals were castrated 5 days after the first injection. Upon castration, there were significant differences in the percent decrease in serum PSA levels (nadir) between animals treated with decoys as compared to the controls. In mock-injected animals, serum levels of PSA dropped by 87% in comparison to precastrate levels, while in decoy AR_1-558_ injected animals the serum levels of PSA dropped by 99% (*p* = 0.0063). A similar result was obtained when GFP-AR_1-558_ chimera proteins were used. GFP injected animals showed a 94% drop in serum levels of PSA compared to GFP-AR_1-558_ that dropped 100% (*p* = 0.0271) (Fig 1A). For patients treated with androgen deprivation, the PSA nadir (lowest PSA reading) is prognostic of the time to develop castration-recurrent disease [6]. Therefore, based upon this data showing that decoys significantly lower the PSA levels (nadir) it is possible that decoys that block AR transcriptional activity may improve prognosis.

The time to castration-recurrence is defined here as the time for serum PSA levels to return to pre-castration levels. The longer the time for serum PSA to reach the precastration level suggests a better prognosis. Mock-injected animals took 7.7 weeks compared to decoy AR_1-558_ injected mice that took 10.3 weeks (*p* = 0.0296) to progress to castration-recurrence. Similarly, GFP injected animals took 9.1 weeks compared to decoy GFP-AR_1-558_ injected mice that took 15.5 weeks (*p* = 0.0245) to progress to castration-recurrence (Fig 1B). The time for PSA to reach pre-castration levels was almost doubled in presence of decoy AR_1-558_ in comparison to control tumors (mock and GFP).

The time for the tumor to double in volume also has prognostic value. The longer it takes for the tumor to double in size, the more favorable the prognosis. In examining this parameter for prognosis, mock-injected animals required 4.1 weeks to double in tumor volume compared to decoy AR_1-558_ injected mice that took 8.4 weeks (*p* = 0.0095). GFP injected animals took 7.1 weeks compared to decoy GFP-AR_1-558_ injected mice that took 11.5 weeks (*p* = 0.021) (Fig 1C). Thus, in the presence of the decoy, approximately twice the amount of time was required for the tumor volume to double in the absence of testicular androgens. Consistent with reduced tumor growth, decoy AR_1-558_ decreased staining of the proliferation marker Ki67 (data not shown) in xenografts as previously reported [2]. Together these data (lower nadir, delayed time for PSA to return to pre-castration levels, and increased time for tumor volume to double) suggest that decoy AR_1-558_ can significantly delay the time for prostate cancer to become castration recurrent.

### Effects of lentivirus delivery of decoy AR_1-558_ on other tissues

**Fig 2:**
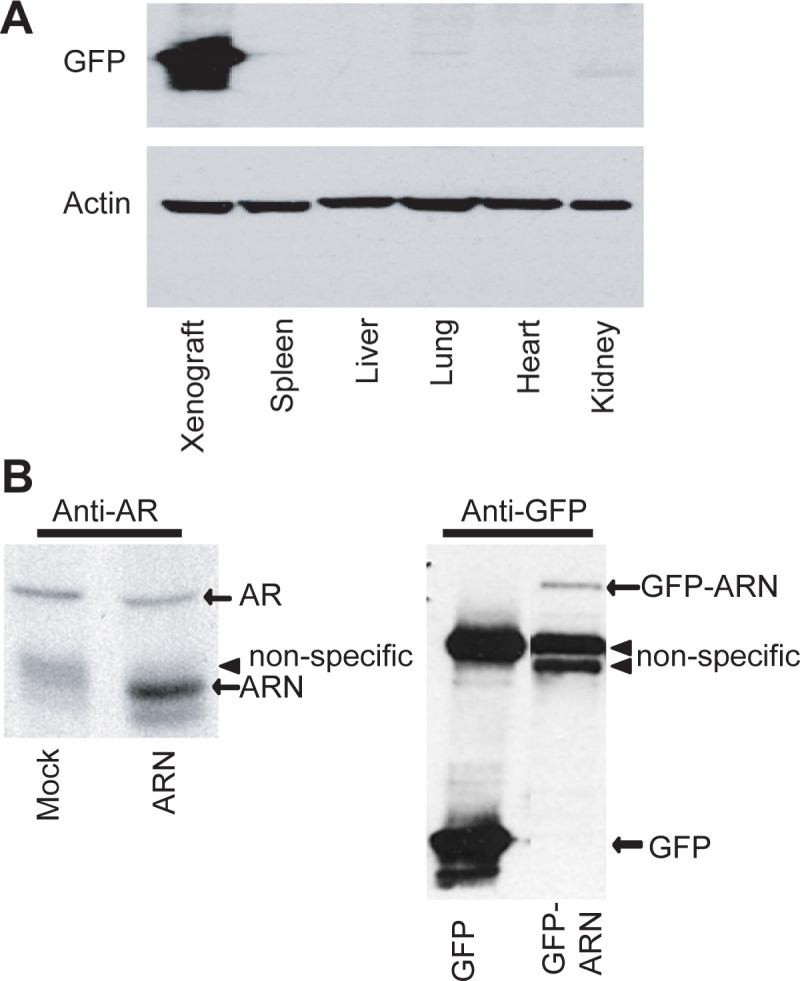
Levels of expression of decoys and endogenous AR *in vivo*. A, Western blot analysis for GFP with a representative animal showing extremely high expression of GFP delivered by lentivirus to the xenograft, yet non-detectable levels of expression in the spleen, liver, lung, heart, and kidney of the same animal. Similar levels of protein (40μg) from whole cell lysates. The membrane was stripped and re-probed for β-actin as a loading control. B, AR, AR_1-558_ (ARN), GFP, and GFP-AR_1-558_ (GFP-ARN) protein levels in harvested xenografts. A non-specific diffuse band migrates slightly slower than AR_1-558_ and was most apparent in the mock-treated lysates.

Upon the duration of the experiment, the major organs were surgically removed for histological review to determine the effect of viral delivery of decoy on spleen, liver, lung, heart, and kidney tissues. H&E staining showed no unusual pathology implying that decoy had no effect on the morphology of these tissues (data not shown). One interpretation could be that AR_1-558_ decoy does not have effect on tissues that are not dependent upon functional AR. Alternatively the lentivirus may not have been delivered to these other organs. To test this, we employed an antibody to GFP and analysed whole cell lysates from these organs from hosts treated with lentivirus. Western blot analysis showed detection of GFP in xenografts harvested from the host (Fig 2A), yet levels of GFP in the organs from the same animals were below levels of detection. This suggests that intratumoral injection of lentivirus to subcutaneous tumors was not efficiently delivered or expressed in other tissues. Since the animals were castrated, we did not examine the murine prostate organ in response to decoy AR_1-558_. Western blot analysis using an antibody to the AR NTD (anti-AR antibody to epitope 299–315) confirmed that decoy AR_1-558_ was delivered to the xenograft (Fig 2B). Levels of endogenous AR were also detected by this antibody, and the levels were not altered by expression of decoy as previously reported [2].

### Decoy AR_1-558_ does not prevent nuclear localization of the AR

**Fig 3:**
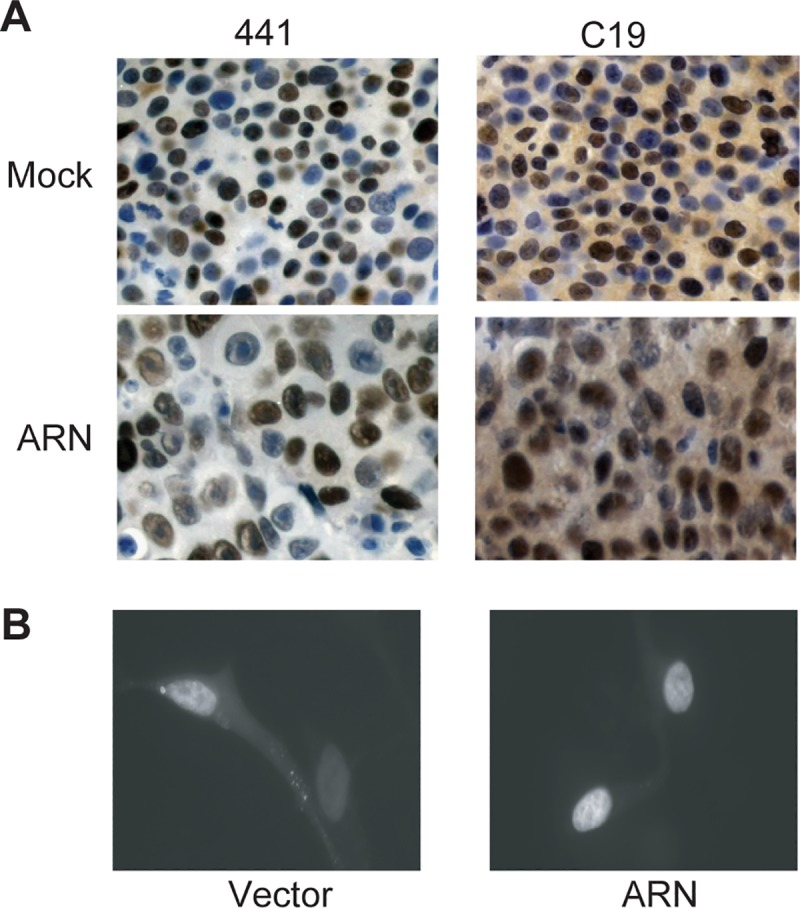
Decoy AR_1-558_ does not prevent nuclear localization of the AR. A, Xenografts were harvested at the duration of the experiments and sections were stained for AR NTD (441) or the LBD (C19). B, Fluorescent microscopy of GFP-AR in LNCaP cells stably expressing vector (left) or decoy (right) and treated with R1881 (10nM) for 30 minutes.

Nuclear AR protein is detected in secondary prostate cancer tumors from patients failing androgen deprivation therapy [7]. To determine if lentivirus delivery of decoys prevented nuclear localization of the AR, xenografts were stained for AR using antibodies that detect both the LBD and the NTD. Staining with an antibody to AR LBD showed predominantly nuclear staining with some cytoplasmic staining regardless of treatment (Fig 3A, column labeled C19). This antibody should only stain the AR and not decoy AR_1-558_. These LNCaP cells do not express detectable levels of AR splice variant protein [8]. An antibody to the NTD stains both decoy and AR and stained the nuclei of cells and was not altered by treatments (column labelled 441). LNCaP cells that stably expressed decoy AR_1-558_, or vector, and treated with androgen for 30 minutes induced the nuclear localization of the GFP-AR regardless of the presence of decoy AR_1-558_ (Fig 3B). Together these data suggest that decoy AR_1-558_ does not prevent nuclear localization of AR.

### Decoy AR_1-558_ reduces the expression of androgen-regulated genes

**Fig 4:**
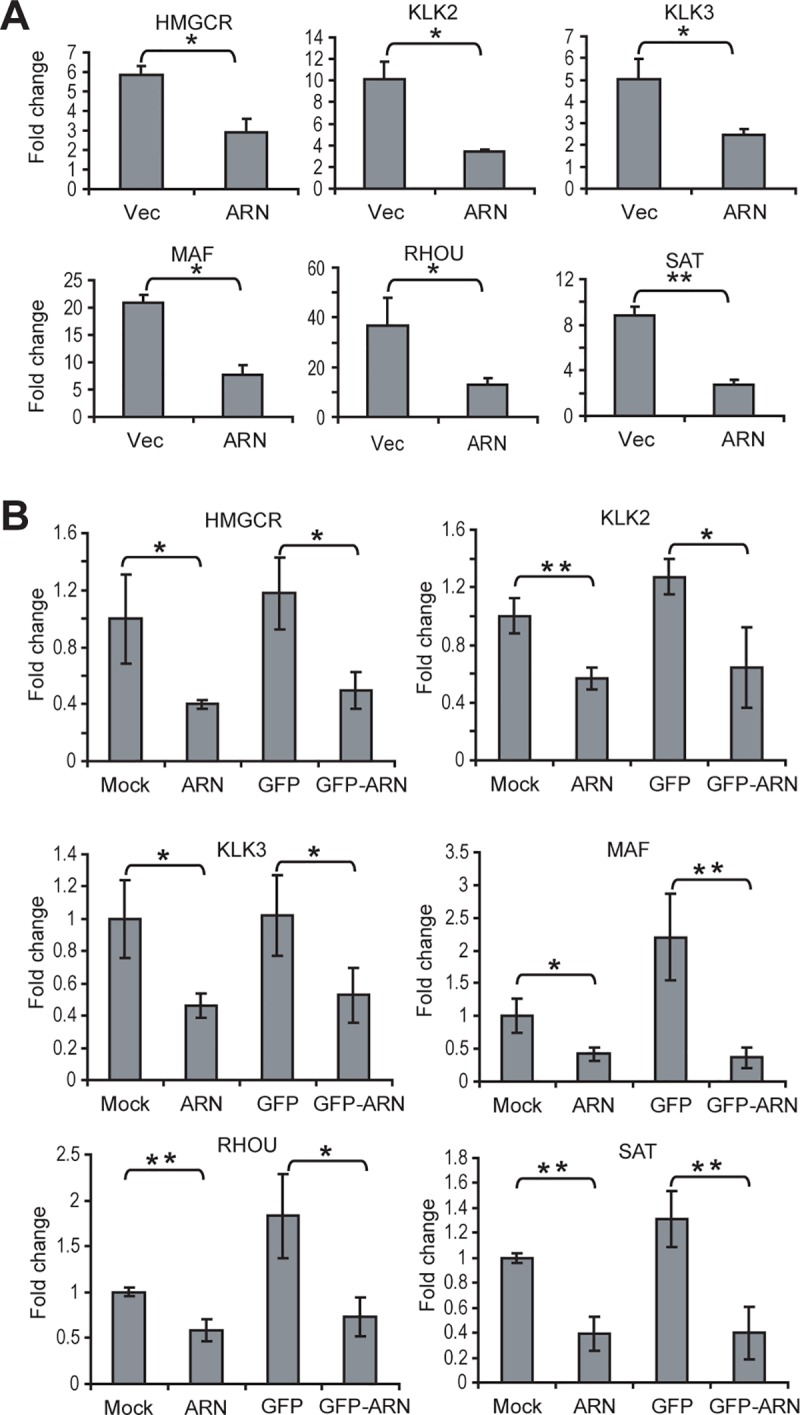
Decoys block the expression of androgen-regulated genes. Real-time qPCR was performed using total RNA isolated from: A, LNCaP cells stably transfected with vector (Vec) or decoys (ARN) and treated for 24 hours with 10nM R1881; or B, xenografts injected with mock, AR_1-558_ (ARN), GFP, and GFP-AR_1-558_ (GFP-ARN). Transcript levels of *HMGCR*, *KLK2*, *KLK3*/PSA, *MAF*, *RHOU*, and *SAT*, were normalized to levels of *GAPDH*. The ratio of each transcript to *GAPDH* is plotted as fold-change. The bars represent the mean ± SD (*n* = 3). Student t-test: * *p*<0.05; **: *p*<0.01.

To determine if decoy AR_1-558_ would alter the expression of androgen-regulated genes, qPCR was performed using total RNA from LNCaP cells that stably express decoy AR_1-558_, as well as from the castration-recurrent xenografts transduced with decoy AR_1-558_. LNCaP cells that stably express vector or decoy were treated with 10 nM R1881 for 24 hours and total RNA was harvested. qPCR was used to measure levels of expression of HMGCR, KLK2, KLK3/PSA, MAF, RHOU, and SAT1 that are normally induced by R1881 [9]. Levels of expression of these genes were significantly decreased in cells stably transfected with decoys (*p*<0.05 or *p*<0.01) in comparison to control cells expressing the vector (Fig 4A). Re-expression of some androgen-regulated genes occurs in castration-recurrent disease [10]. Therefore, we also measured the expression of the same set of genes using RNA prepared from harvested LNCaP tumors from castrated mice that had been treated lentivirus for decoys or controls (described in Fig 1). Levels of expression of these androgen-regulated genes that were decreased by decoys *in vitro* were also decreased *in vivo* in castration-recurrent tumors transduced with decoys (Fig 4B). These data suggest that decoy AR_1-558_ interferes with the regulation of androgen-regulated genes both *in vitro* in cells treated with R1881 and *in vivo* in castration-recurrent xenografts. Together, these *in vitro* and *in vivo* results support that decoy AR_1-558_ inhibits the transcriptional activity of endogenous AR.

### Decoy AR_1-558_ blocks AR-ARE interactions

**Fig 5:**
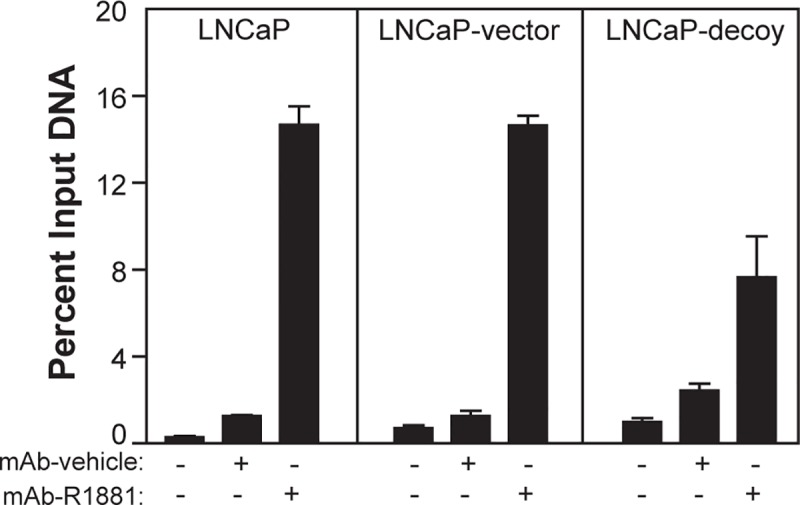
Decoys block AR-ARE interaction. ChIP was performed in non-transfected and stably transfected (vector or decoy) cells in response to androgen. Parental LNCaP cells (untransfected) or stables expressing vector or decoy were treated ± androgens (R1881, 10 nM) for 3 hours and used in ChIP analyses with rabbit IgG (no antibody negative control) and anti-AR (mAb; C19 antibody to AR LBD). Eluted DNA fragments were purified and used for qPCR with primers designed to amplify the PSA ARE. Bars show the percentage input as the mean ± SD (*n* = 3). A representative result from repeated experiments is shown.

Gene expression data generated here from *in vitro* and *in vivo* experiments provided evidence that decoy AR_1-558_ had a direct effect on the transcriptional activity of AR. To determine if decoy AR_1-558_ potentially altered the formation and stabilization of the transcriptional complex on AREs, we performed ChIP assays. Interaction of endogenous AR with the ARE in the enhancer of the PSA gene was measured in non-transfected and stably transfected (vector or decoy) cells in response to androgen. These studies revealed that the decoys significantly (*p*<0.05) reduced AR-ARE interaction by 50% in presence of R1881 in cells stably expressing the decoys in comparison to cells untransfected or solely expressing the vector (Fig 5**)**. These data were consistent with reduced expression of PSA mRNA in cells expressing the decoy AR_1-558_ (Fig 4). Thus, decoy AR_1-558_ reduced interaction of endogenous AR with AREs which would result in decreased levels of transcription of androgen-regulated genes.

### AR NTD decoys do not interact with the AR

**Fig 6:**
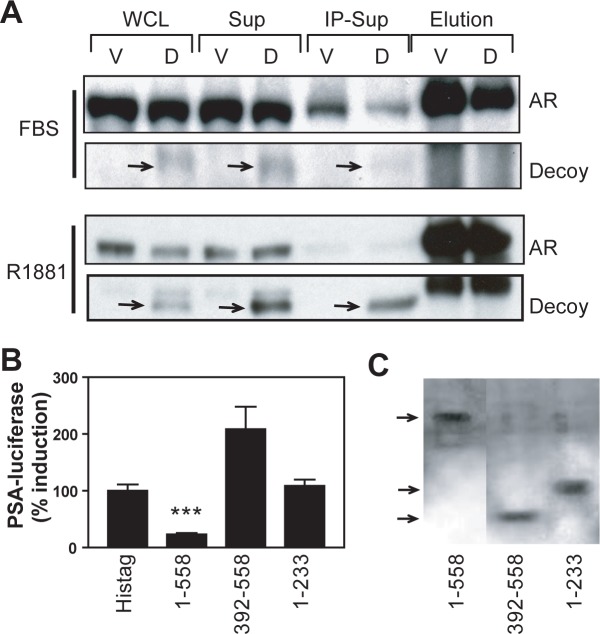
Decoy molecules do not interact with the AR. A, LNCaP cells stably expressing vector (V) or decoy AR_1-558_ (D) were incubated in serum (FBS) or with R1881 (1nM) for 3 h followed by immunoprecipation of the AR using an antibody to the LBD (Santa Cruz C19). Whole cell lysates (lanes 1 and 2), supernatant (lanes 3 and 4), wash (lanes 5 and 6), and immunoprecipitated complex-IP elution (lanes 7 and 8) were analyzed by Western blot using antibody to the AR NTD (Santa Cruz 441) to detect both AR and the decoy AR_1-558_. B, Decoy AR_1-558_ blocked ligand-independent activation of the AR by forskolin while AR_1-233_ and AR_392-558_ did not. LNCaP cells were transiently transfected with PSA(-630/+12)-luciferase reporter and expression vectors for His-tag, His-AR_1-558_, His-AR_1-233_, and His-AR_392-558_ and treated with forskolin (50μM) for 48 h under serum-free conditions. The percent induction of PSA-luciferase activities relative to values achieved with expression of His-tag is shown. Bars represent the mean ± SE of three separate experiments. C, Western blot analysis using an antibody to His-tag with whole cell lysates from LNCaP cells transiently transfected with expression vectors for His-tag, His-AR_1-558_, His-AR_1-233_, and His-AR_392-558_.

To initiate transcription in response to androgen, dimerization of AR is needed and requires interactions between DNA-binding domains (i.e., DBD/DBD) (for a review see [11]). Heterodimerization of AR with truncated AR splice variant AR-V7 that lacks LBD and homodimerization of AR-Vs also requires DBD/DBD interactions [12]. Here the decoy lacks a DBD and is not predicted to interact with AR because of the requirement of DBD/DBD interactions. However, since ^23^FQNLF^27^ and ^429^WHTLF^433^ in AR NTD can be important in AR NTD-LBD interaction in response of AR to androgen [13], we tested if AR interacted with decoy. To do this, we immunoprecipated the AR using an antibody to the LBD of the AR in LNCaP cells that stably expressed the decoy. Cells used were either continually passaged in whole serum, or had been serum-starved prior to treatment with R1881 for 3 hr. Western blot analyses using an antibody to the NTD detected both decoy and the FL-receptor in samples prepared from whole cell lysates, supernatant, and wash (Fig 6A). Cells without decoy (stably transfected with vector) had no protein bands at the expected MW for the decoy (compare vector lanes to decoy lanes). Decoy AR_1-558_ was not detected in immunoprecipitated complexes with the AR (see lanes 7 and 8). This suggests that decoy AR_1-558_ does not interact with the AR to inhibit the receptor through a dominant negative mechanism.

To determine if decoys that contain ^23^FQNLF^27^ or ^429^WHTLF^433^ could repress expression of PSA, we co-transfected LNCaP cells with expression vectors encoding regions with amino acids 1–233 (contains ^23^FQNLF^27^) or 392–558 (contains ^429^WHTLF^433^) of the AR NTD and PSA promoter-luciferase reporter gene construct prior to treating the cells with forskolin. Consistent with previous reports, forskolin increased PSA-luciferase activity in the absence of serum and androgen [2,14]. Decoy AR_1-558_ significantly reduced the induction of PSA-luciferase activity compared to control (positive control labeled “His-tag”) as shown previously [2]. Decoys encoding amino acids 1–233 or 392–558 that contain the (F/W)XXLF motifs did not inhibit induction of PSA-luciferase activity (Fig 6B). Levels of expression of these constructs were similar in cells as shown by Western blot analysis using an antibody to the his-tag motif (Fig 6C). Thus, the differences between the constructs of the AR NTD to inhibit PSA-luciferase activity was not due to reduced levels of expression. Together with the lack of detection of interaction between decoy AR_1-558_ and AR by co-immunoprecipitation, these data do not support a dominant negative mechanism of decoy AR_1-558_ to inhibit transcriptional activity of AR.

**Fig 7:**
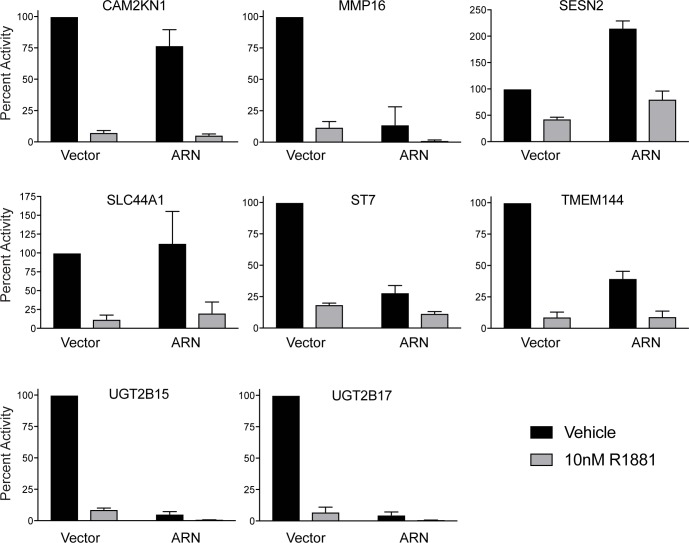
Effects of decoy on androgen-repressed genes. Real-time qPCR was performed using total RNA isolated from LNCaP cells stably transfected with vector or decoy (ARN) and treated for 24 hours with 10nM R1881. Transcript levels of *CAM2KN1*, *MMP16*, *SESN2*, *SLC44A1*, *ST7*, *TMEM144*, *UGT2B15*, and *UGT2B17* were normalized to levels of *GAPDH*. The ratio of each transcript to *GAPDH* is plotted as percent activity relative to vector control. The bars represent the mean ± SD (*n* = 3).

Androgens increase as well as repress gene expression through the full-length AR. Antiandrogens can de-repress the expression of some genes turned off in response to androgens. Here, in the presence of androgen, decoy AR_1-558_ did not de-repress the expression of the known androgen-repressed genes that were tested (Fig 7).

## Discussion

Androgen deprivation therapy causes a temporary reduction in tumor burden concomitant with a decrease in serum levels of PSA [15] which is an AR-regulated gene. Unfortunately, prostate cancer will eventually begin to grow again in the absence of androgens to form castration-recurrent disease as characterized by a rising titer of serum PSA [16]. Enzalutamide and abiraterone, initially reduce serum PSA and increase survival by approximately 6 months in castration-recurrent patients [17,18]. Resistance to these drugs is associated with increasing serum levels of PSA which implies re-activation of AR transcriptional activity.

Evidence supporting the *in vivo* efficacy of targeting the AR NTD was shown by application of decoy AR_1-558_ molecules of AR NTD that inhibit the growth and hormonal progression of prostate cancer xenografts both in the presence and absence of androgen [2]. Those studies applied two models: one model used cells that stably expressed decoy AR_1-558_ to grow tumors with decoy; and the other model applied lentivirus delivery of decoy to established tumors in non-castrated mice. Here we investigated the effects of decoy in established tumors combined with castration. This is a model that better reflects the clinical representation of patients that undergo androgen deprivation therapy and develop castration-recurrent disease. Non-transfected LNCaP tumors were first established in NOD-SCID mice prior to castration and lentivirus delivery of the decoys. PSA nadir, time for PSA to reach pre-castration levels, and the time for the tumor to double in volume after therapy were all measured as an indication of tumor progression as reported in clinical studies [19,20]. Patients have a better prognosis when their PSA nadir reaches very low levels, or with long periods of the time required for rises in PSA or for their tumor volume to double. Our studies showed that the delivery of decoys to established tumors leads to a lower nadir, increased time for the tumor to double in volume as well as for PSA to reach the pre-castrate levels. These data concur with delayed hormonal progression in xenografts of LNCaP cells stably expressing decoy in castrated mice [2] and suggests that tumor progression is at least in part dependent on the AR NTD.

AR NTD has a high degree of intrinsic disorder and acts as a hub for protein-protein interactions. Not surprising is also the fact that the NTD is highly posttranslationally modified which can alter protein interactions and localization. Within the NTD is AF-1 which is essential for transcriptional activity. Thus targeting AR NTD for therapeutic intervention would be effective in preventing its transcriptional activity regardless of the presence or absence of ligand and LBD. Here we show that in combination with castration, lentivirus delivery of decoys to the AR NTD to established tumors delayed hormonal progression to castration-recurrence. The mechanism of action of the decoys involved decreased expression of genes normally regulated by androgen, and reduced physical interaction of the AR with AREs. The decoys did not reduce levels of endogenous AR protein, nor did decoys inhibit the activity of the AR through a dominant negative mechanism or involve amino acids 1–233 or 392–558 which contain the (F/W)XXLF motifs as well the core sequences for Tau-1 (^178^LKDIL^182^) and Tau-5 (^435^WHTLF^439^). The decoys also did not prevent nuclear localization of AR in response to ligand. Thus, overexpression of decoy AR_1-558_ allows the AR to be in the nucleus yet still prevents the interaction of endogenous AR with AREs to reduce transcription of genes regulated by AR.

Potentially, the mechanism by which decoy AR_1-558_ inhibits AR may involve changes in protein-protein interactions required for a stable and functional transcriptional complex. These interactions may involve coactivators, kinases, or enzymes altering posttranslational modifications. Currently, the AR is suggested to interact with over 169 different proteins [21], thereby complicating the identification of proteins that may be blocked by the decoys. However, decoy AR_1-558_ had no effect on PC3 xenografts that do not express AR, suggesting that decoy AR_1-558_ does not mop-up critical proteins that are generally required for survival and growth [2]. Although the AR NTD shares less than 15% homology with progesterone receptor (PR), glucocorticoid receptor (GR) and estrogen receptor (ER), these receptors do interact with some of the same proteins (e.g., SRC-1). Previous work has shown that decoy AR_1-558_ does not inhibit the transcriptional activity of ER or GR, suggesting it does not compete with proteins that are limiting for the activities of these transcription factors. However, PR was significantly inhibited by decoy AR_1-558_ [2]. These data highlight potential molecular mechanisms shared between the AR and PR. Recently, PR has been reported to be highly expressed and an independent negative prognostic factor for clinical trials for prostate cancer [22] thereby making inhibition of PR a potential benefit of an approach that blocks both AR NTD and PR.

Repression of gene expression by androgen may involve many different mechanisms and different domains of the androgen receptor. Corepressors such as SMRT can bind the AR NTD to mediate transrepression [23]. The mechanism of androgen repression of cyclin D1 gene expression involves androgen-bound AR recruited to a negative ARE and an SP1-binding site with recruitment of a repression complex that includes DAX-1 and HDAC-1 [24]. Some other repression mechanisms elucidated involve interaction of AR DNA-binding domain with Sp-1 or AR interactions with other proteins such as Runx2 protein [25], SF-1 [26], ATF-2 [27], FOXO1 [28], HOXB13 [29], and lysine-specific demethylase 1 (LSD1) [30]. Genes tested here are known to be repressed by androgen and MMP16, SLC44A1, ST7, and UGT2B15 and UGT2B17 all have AR binding sites [31,32]. However, these AREs are not necessarily negative AREs since UGTY2B15/B17 requires AR for basal expression as well [32]. If AR is knocked down, levels of UGT2B15/17 are attenuated [32] thereby highlighting the complexity of the mechanism. If decoy AR_1-558_ does not bind to the full-length AR, but rather competes with full-length AR for essential coactivators or other proteins that bind to the AR NTD as we suggest to inhibit transactivation, it may be that the mechanism of androgen repression does not involve AR NTD, at least not for these specific genes. Further experiments are warranted to delineate the mechanisms of how antagonists of AR NTD impact androgen-repressed genes.

In conclusion, our findings here support that the AR NTD is a feasible therapeutic target for the development of novel drugs for the treatment of prostate cancer. Recently the first AR NTD antagonist, a prodrug of EPI-002 [33,34], started Phase 1 clinical trials for prostate cancer patients that have failed abiraterone and/or enzalutamide (Clinical trials NCT02606123).

## Supporting information

S1 Fig Uncut gel of Fig 6C. (PPTX)
