# Supplementary figures and images for: Inhibition of androgen receptor by decoy molecules delays progression to castration-recurrent prostate cancer
Source: PLoS One. 2017 Mar 17;12(3):e0174134. doi: 10.1371/journal.pone.0174134 (PMC5357013; doi:10.1371/journal.pone.0174134)

## Slide 1
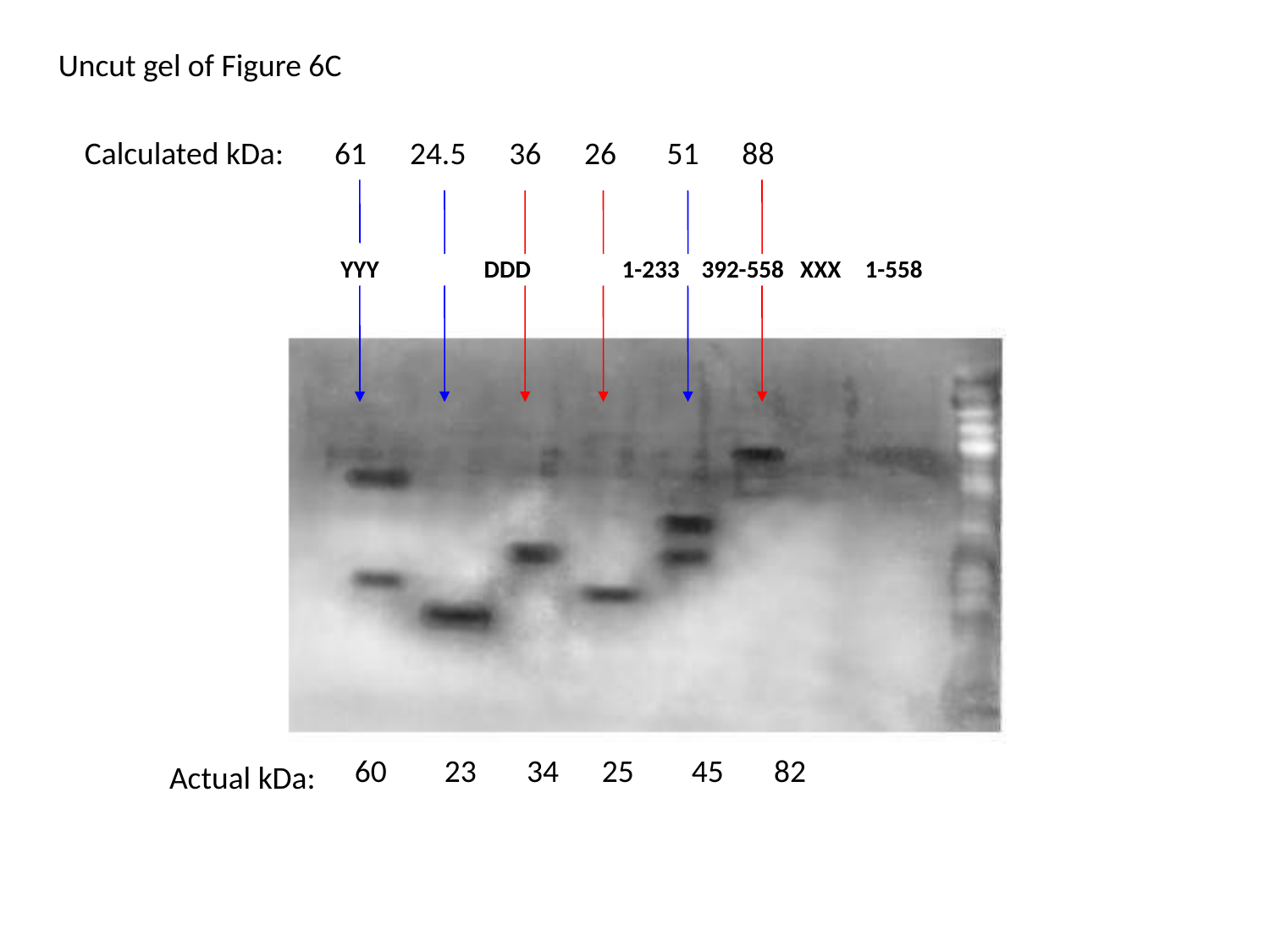

Uncut gel of Figure 6C
Calculated kDa:
 61 24.5 36 26 51 88
YYY	 DDD	 1-233 392-558 XXX	 1-558
60 23 34 25 45 82
Actual kDa:

Supplement: S1 Fig — (PPTX) [file pone.0174134.s001.pptx]
